# Editorial: Women in science: public health education and promotion 2023

**DOI:** 10.3389/fpubh.2024.1368704

**Published:** 2024-02-12

**Authors:** Sunjoo Kang, Zsuzsa Rákosy, Se Eun Park, Thi Anh Phuong Nguyen

**Affiliations:** ^1^Department of Global Health and Disease Control, Graduate School of Public Health, Yonsei University, Seoul, Republic of Korea; ^2^Department of Public Health Medicine, Medical School, University of Pécs, Pécs, Hungary; ^3^Bethesda Children's Hospital, Budapest, Hungary; ^4^International Vaccine Institute, SNU Research Park, Seoul, Republic of Korea; ^5^Faculty of International Education, Hue University of Medicine and Pharmacy, Hue University, Hue, Vietnam

**Keywords:** women in science, public health, education, health promotion, health literacy, health disparity

Academic research dedicated to developing effective educational programs is increasingly intertwining with the pivotal role of health literacy in healthcare policies. The World Health Organization (WHO) emphasizes that tailored health literacy initiatives are fundamental in combating non-communicable diseases and health disparities (1, 2). Recent responses to emerging infectious diseases have underscored the urgency of addressing health literacy gaps within marginalized and underserved communities, given the proliferation of misinformation (3). This urgency is compounded by persistent disparities in ethnic minority vaccine access (2) and variations in health literacy levels (4, 5), necessitating tailored solutions that factor in diverse population characteristics.

On a practical front, there's an immediate and indispensable need to fortify governance structures and foster collaboration among diverse stakeholders within the healthcare sector. This imperative seeks to unearth governance models that can maximize the impact of health policies and education, serving as the bedrock for sustainable progress in public health practices (6, 7).

The Research Topic meticulously highlighted the significant contributions of women researchers in Public Health, especially within the realms of Education and Promotion. It not only celebrated the exceptional achievements of female researchers but also underscored the pivotal role played by studies led by women in public health education and promotion. These articles, led by female first or last authors, fostered collaborative efforts between early career researchers and their senior female colleagues. This editorial aims to provide a comprehensive overview of the 2023 Research Topic on *Women in Science – Public Health Education and Promotion*. The published research articles significantly enrich our understanding of public health dynamics, each presenting a unique facet of public health and collectively advancing knowledge in this field. Furthermore, these research endeavors distinctly underline the invaluable role of women researchers in driving progressive insights and solutions within the public health domain. The authors' work is organized and summarized according to article type below.

## Original research

The focus on health literacy interventions sheds light on their impact and offers more profound insights into the broader implications of health education. Understanding the intricate relationship between health literacy and learner motivation is a potential game-changer in enhancing educational outcomes and fostering improved health practices. Moreover, a comprehensive exploration of menstrual health research holds promise in advancing critical aspects of women's health, potentially shaping policy and educational initiatives in this vital public health domain. Bíró et al. investigated health literacy's multifaceted impact on behaviors, vaccination confidence, and healthcare usage. Their comprehensive study uncovered a nuanced relationship between health literacy dimensions and various health aspects. Despite no consistent link found between health literacy and behavior, sporadic effects of digital health literacy (DHL) on healthcare usage surfaced. Interestingly, both general health literacy (GHL) and DHL showed positive correlations with self-perceived health and vaccination confidence. Prioritizing enhancement in both dimensions holds promise in significantly improving self-perception and fostering vaccination confidence, suggesting a vital need for interventions to bolster GHL before fortifying DHL. The implications underscore the importance of elevated health literacy levels in mitigating health disparities, particularly pivotal for future vaccination campaigns.

Research by Malik et al. examined menstrual experiences and attitudes toward menstrual hygiene among women in Pakistan. The study highlighted a stark contrast between high awareness of menstrual hygiene and limited knowledge regarding health conditions associated with abnormal menstrual cycles. Their findings emphasized the need for comprehensive menstrual health education to bridge knowledge gaps. Furthermore, the success of FemPure products illustrated a strong demand for organic, user-friendly menstrual products, underlining the necessity for policy interventions to drive the development and accessibility of suitable menstrual products. The implications point toward an urgent requirement to destigmatize discussions on menstrual health and effectively address societal taboos through education and policy reforms.

Mózes et al. investigated screening attendance disparities among Hungarian-speaking Roma and non-Roma women across Hungary, Romania, and Slovakia, uncovering crucial factors like chronic diseases, smoking, health insurance, and physical inactivity contributing to attendance disparities. These revelations underline the urgent need for targeted screening programs to reduce healthcare discrepancies. The prominence of lacking insurance as a pivotal barrier Accentuates the urgency for comprehensive coverage to ensure equitable healthcare access. The implications resonate with the necessity of tailored screening initiatives, particularly within the Roma community, to enhance preventive healthcare measures and bridge existing healthcare gaps.

Rachubińska et al. examined the intricate role of depression as a mediator between personality traits and work addiction among women in Poland. Their study revealed correlations between neuroticism, extraversion, agreeableness, conscientiousness, and work addiction, mediated by depression, highlighting the significance of addressing depressive symptoms in managing workaholism. These findings stress the immediate need for tailored interventions and robust workplace support systems aimed at effectively mitigating work addiction tendencies. The study strongly advocates the implementation of comprehensive mental health interventions within work settings to foster healthier and more supportive work environments.

## Perspectives

Caron et al. highlighted disparities in public health education among U.S. academic institutions, particularly in Historically Black Colleges and Universities (HBCUs). Identifying significant gaps in public health curricula and educational pathways accentuates the urgency for standardizing and expanding public health education. These enhanced curricula aim to equip individuals to effectively address pressing public health challenges, especially in crises like the ongoing COVID-19 pandemic, preparing future generations to manage diverse public health crises with proficiency.

## Conclusion

This research showcases the global contributions of women in the field of public health education and promotion. It encompasses general perspectives inspired and initiated by women in a specific research field, articles celebrating outstanding female researchers, and research studies led by women investigating public health education and/or health promotion under the Women in Public Health Education and Promotion, 2023 collection. This collection emphasizes women's contributions and focuses on the significance of women's research and advancements in public health, underscoring their pivotal role in this domain.

## Author contributions

SK: Writing – original draft, Writing – review & editing. ZR: Writing – original draft, Writing – review & editing. SP: Writing – original draft, Writing – review & editing. TN: Writing – original draft, Writing – review & editing.
